# The type 2 diabetes-associated HMG20A gene is mandatory for islet beta cell functional maturity

**DOI:** 10.1038/s41419-018-0272-z

**Published:** 2018-02-15

**Authors:** Jose M. Mellado-Gil, Esther Fuente-Martín, Petra I. Lorenzo, Nadia Cobo-Vuilleumier, Livia López-Noriega, Alejandro Martín-Montalvo, Irene de Gracia Herrera Gómez, Maria Ceballos-Chávez, Laura Gómez-Jaramillo, Antonio Campos-Caro, Silvana Y. Romero-Zerbo, Júlia Rodríguez-Comas, Joan-Marc Servitja, Gemma Rojo-Martinez, Abdelkrim Hmadcha, Bernat Soria, Marco Bugliani, Piero Marchetti, Francisco J. Bérmudez-Silva, Jose C. Reyes, Manuel Aguilar-Diosdado, Benoit R. Gauthier

**Affiliations:** 1 Department of Cell Regeneration and Advanced Therapies, Andalusian Center of Molecular Biology and Regenerative Medicine-CABIMER, Junta de Andalucia-University of Pablo de Olavide-University of Seville-CSIC, Seville, Spain; 2 Department of Genome Biology, Andalusian Center of Molecular Biology and Regenerative Medicine (CABIMER) JA-CSIC-UPO-US, Seville, Spain; 3 Research Unit, University Hospital “Puerta del Mar”, Instituto de Investigación e Innovación en Ciencias Biomédicas de la Provincia de Cádiz (INiBICA), Cádiz, Spain; 4 Unidad de Gestión Clínica Intercentros de Endocrinología y Nutrición, Instituto de Investigación Biomédica de Málaga (IBIMA), Hospital Regional Universitario de Málaga, Universidad de Málaga, Málaga, Spain; 5 Diabetes & Obesity Research Laboratory, Biomedical Research Institute August Pi I Sunyer (IDIBAPS), Barcelona, Spain; 6 Centro de Investigación Biomédica en Red de Diabetes y Enfermedades Metabólicas Asociadas (CIBERDEM), Madrid, Spain; 7 Department of Translational Research and of New Surgical and Medical Technologies, University of Pisa, Pisa, Italy; 8 Endocrinology and Metabolism Department University Hospital “Puerta del Mar”, Instituto de Investigación e Innovación en Ciencias Biomédicas de la Provincia de Cádiz (INiBICA), Cádiz, Spain

## Abstract

HMG20A (also known as iBRAF) is a chromatin factor involved in neuronal differentiation and maturation. Recently small nucleotide polymorphisms (SNPs) in the *HMG20A* gene have been linked to type 2 diabetes mellitus (T2DM) yet neither expression nor function of this T2DM candidate gene in islets is known. Herein we demonstrate that *HMG20A* is expressed in both human and mouse islets and that levels are decreased in islets of T2DM donors as compared to islets from non-diabetic donors. In vitro studies in mouse and human islets demonstrated that glucose transiently increased *HMG20A* transcript levels, a result also observed in islets of gestating mice. In contrast, HMG20A expression was not altered in islets from diet-induced obese and pre-diabetic mice. The T2DM-associated rs7119 SNP, located in the 3′ UTR of the *HMG20A* transcript reduced the luciferase activity of a reporter construct in the human beta 1.1E7 cell line. Depletion of *Hmg20a* in the rat INS-1E cell line resulted in decreased expression levels of its neuronal target gene *NeuroD* whereas *Rest* and *Pax4* were increased. Chromatin immunoprecipitation confirmed the interaction of HMG20A with the *Pax4* gene promoter. Expression levels of *Mafa*, *Glucokinase*, and *Insulin* were also inhibited. Furthermore, glucose-induced insulin secretion was blunted in HMG20A-depleted islets. In summary, our data demonstrate that HMG20A expression in islet is essential for metabolism-insulin secretion coupling via the coordinated regulation of key islet-enriched genes such as *NeuroD* and *Mafa* and that depletion induces expression of genes such as *Pax4* and *Rest* implicated in beta cell de-differentiation. More importantly we assign to the T2DM-linked rs7119 SNP the functional consequence of reducing *HMG20A* expression likely translating to impaired beta cell mature function.

## Introduction

Type 2 Diabetes Mellitus (T2DM) is a metabolic disease characterized by impaired insulin secretion and/or action in target organs that leads to elevations in blood glucose levels. Environmental factors as well as strong genetic components contribute to the pathogenesis of T2DM. Thus far, 100–120 susceptibility loci have been associated to T2DM by Genome Wide Association Studies (GWAS)^1–3^. Although functional defects remain to be assigned, many of these loci point to primary defects in beta cell function rather than to insulin resistance^4^. In this context, several SNPs within the *HGM20A* gene (also known as *iBRAF*) have been associated to T2DM^5–8^. Of particular interest is the rs7119 SNP located within the 3′ UTR of the *HMG20A* transcript. In silico analysis revealed that rs7119 modifies a functional microRNA (miRNA) cis-regulatory element^9^. However, the molecular consequences of this SNP on the expression of *HMG20A* and/or its regulation and impact on islet physiology are still unknown.

*HMG20A* is a member of the high mobility group (HMG) box-containing genes that binds to chromatin and exerts global genomic changes through establishing active or silent chromatin^10^. HMG20A is highly expressed in mature neurons and plays a key role in promoting neuronal differentiation during development^11^. In this context, HMG20A relieves the transcriptional repression imposed by the complex LSD1–CoREST histone demethylase^12^, which function is to silence neuronal genes in non-neuronal tissues through its interaction with the transcription factor REST. In analogy, epigenetic repression of the *Rest* gene in pancreatic precursors was shown to coincide with the activation of the core beta cell program^13^. Mature pancreatic beta cells do not express REST while forced expression results in inhibition of neuronal proteins of the insulin exocytotic machinery such as SNAP25 and SYNAPTOTAGMIN VII (SYT7) leading to impaired glucose-induced insulin secretion^14^. Interestingly, REST was shown to repress expression of key beta cell development genes, such as *NEUROD* and *PAX4*^15,16^. *NEUROD* is a bona fide target gene of HMG20A in neuronal cells^11^ and mutations in this gene predispose individuals to maturity onset diabetes of the young 6 (MODY6)^17^.

HMG20A is also implicated in epithelial-to-mesenchymal transition (EMT) through interaction with specific key regulators of this process such as SNAIL^18^. EMT is an example of cell plasticity and a key process during embryonic development, and together with the reverse transformation, the mesenchymal-to-epithelial transition (MET), are required for the formation of organs in the final destinations of embryonic migratory cells^19,20^. Both processes occur during pancreatic and islet development and require extensive reorganization of the epigenetic information of the cells. Remarkably, not only in development but also in response to different physiological demands, beta cells may de-differentiate in order to acquire plasticity capabilities and increase survival^21,22^, two processes that may implicate PAX4^23,24^.

Based on these findings, we hypothesized that HMG20A may contribute to the regulation of key genes such as *NeuroD* and *Pax4* which are essential for beta cell functional maturity as well as survival. Towards this goal, herein we investigate the expression profile and the gene regulatory function of HMG20A in pancreatic islets, as well as determine whether the SNP rs7119 associated with T2DM impacts HMG20A expression. We report that T2DM-associated SNP rs7119 leads to altered HMG20A expression, and that HMG20A regulates metabolism-secretion coupling genes as well as functional maturity of beta cells.

## Results

### *HMG20A* is expressed in pancreatic islets and transcript levels are decreased in islets from T2DM donors

**Fig. 1:**
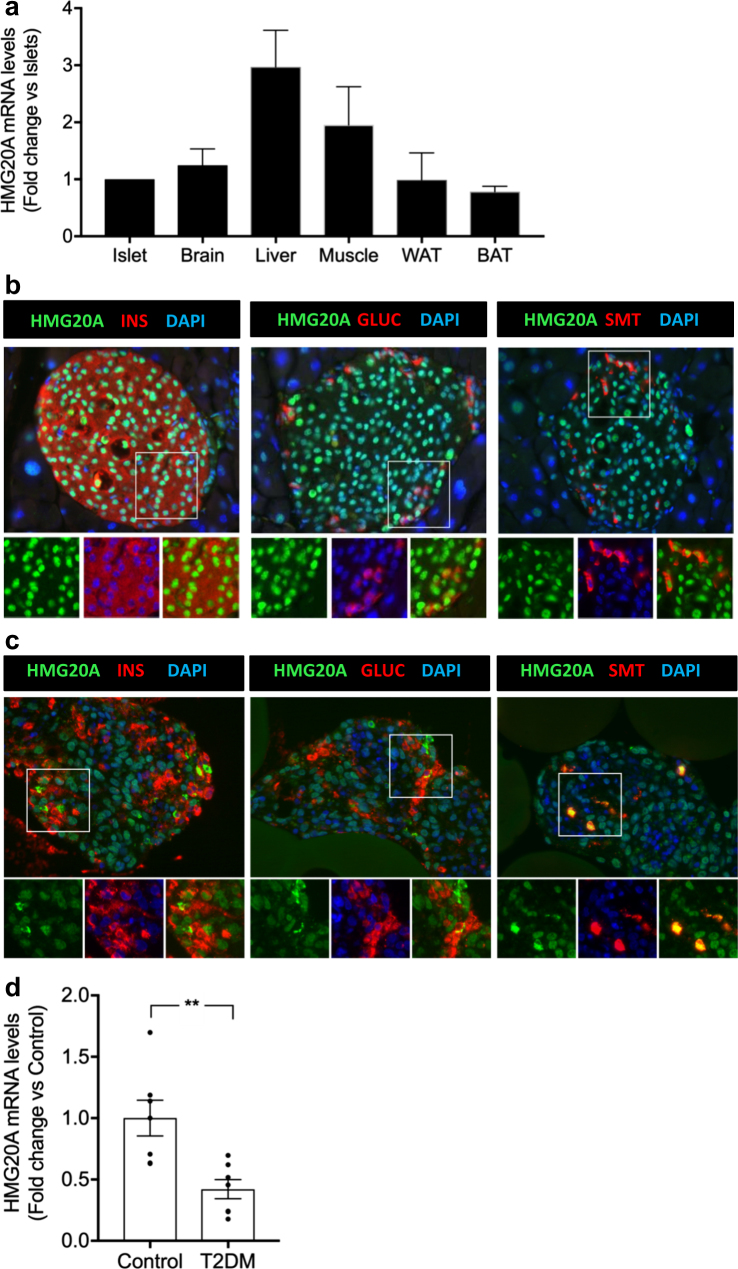
HMG20A is expressed in both mouse and human islets and is decreased in islets from T2DM donors. **a** HMG20A transcript levels were assessed by qPCR in islets, brain, liver, muscle, white adipose tissue (WAT), and brown adipose tissue (BAT) from mice (*n* = 6). Representative images of **b** mouse and **c** human islets co-stained for HMG20A (green) along with INSULIN (INS), GLUCAGON (GLUC) or SOMATOSTATIN (SMT) (red). Nuclei are stained using DAPI (blue). Magnification 40×. White boxes define areas enlarged in panels bellow. **d** *HMG20A* mRNA levels were measured by qPCR in human islets isolated from normoglycemic (control) or type 2 diabetic (T2DM) organ donors (*n* = 7). Data are depicted as dot plots with means ± S.E.M. *p* values were determined using unpaired two-tailed Student's *t*-test. **p < 0.01

As a first step to assign a potential role of the *HMG20A* gene in pancreatic islet physiology, we determined its transcript levels in islets as compared to other tissues. Mouse pancreatic islets displayed comparable expression levels of *Hmg20a* to other organs such as adipose tissue (white and brown), brain and muscle whereas the liver displayed highest levels (Fig. 1a). HMG20A co-stained with INSULIN (beta cells), GLUCAGON (alpha cells) and SOMATOSTATIN (delta cells) whereas its expression was rarely detected in exocrine pancreas (Fig. 1b). A similar endocrine cell expression pattern was detected in human islets (Fig. 1c). We next assessed whether expression levels of *HMG20A* were altered in islets isolated from T2DM donors. The rational was to establish a correlation between *HMG20A* levels and the hyperglycemic environment that may be altered by T2DM-linked SNPs. *HMG20A* transcript levels were decreased by ~60 % in islets from T2DM patients as compared to islets purified from normoglycemic control donors (Fig. 1d).

### Metabolic stressors modulate *HMG20A* expression in islets

**Fig. 2:**
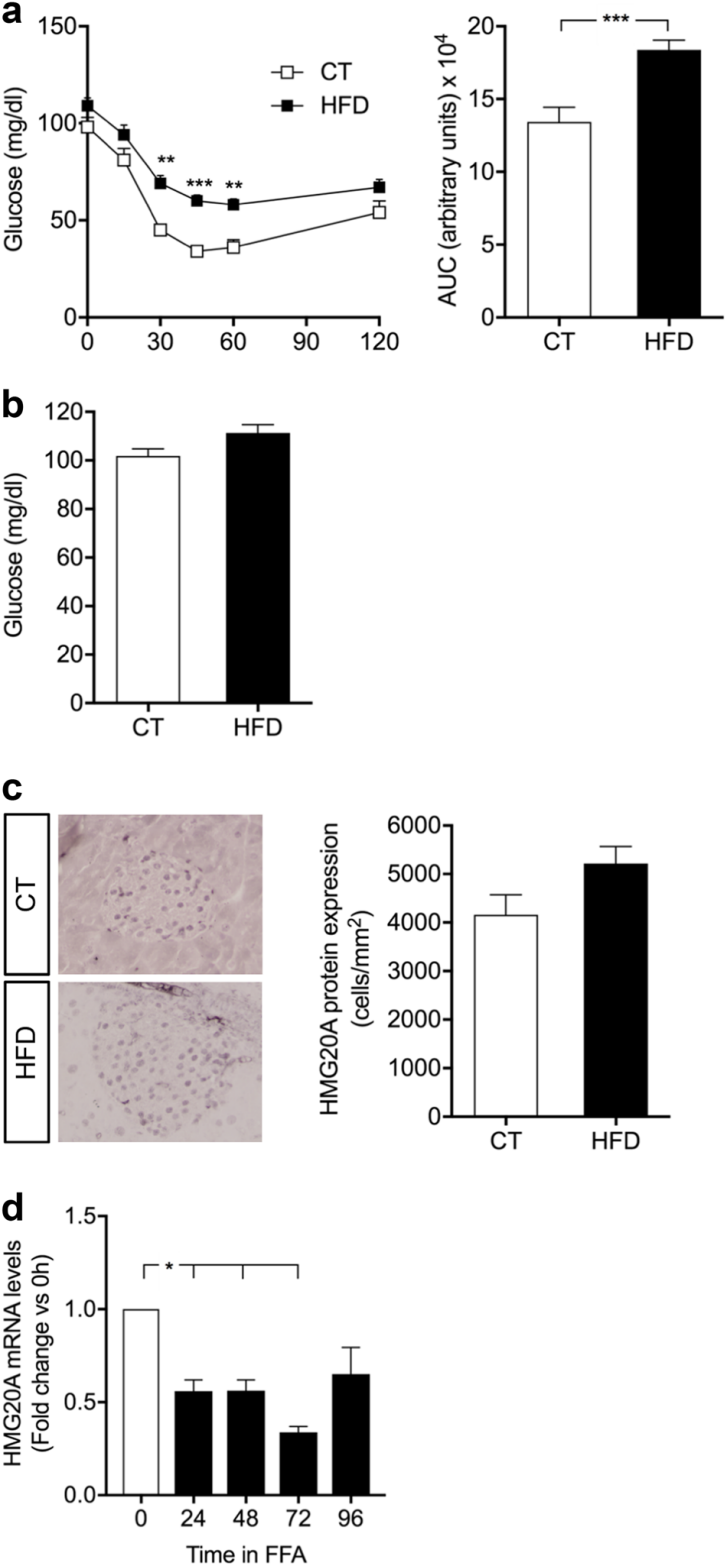
HMG20A protein expression is not altered in islets of high fat diet (HFD)-induced obesity and pre-diabetic mice. **a** Insulin tolerance test (ITT) and area under the curve (AUC) analysis in both control (CT) and HFD mice (CT, *n* = 10; HFD, *n* = 15). **b** Fasting blood glucose levels in both control and HFD mice (CT, *n* = 10; HFD, *n* = 15). **c** Representative images of pancreatic sections from HFD and CT mice stained and quantified for HMG20A (*n* = 4). **d** *Hmg20a* mRNA levels were assessed by qPCR in INS-1E cells cultured with 0.05 mM palmitate for 24, 48, 72 or 96 h (*n* = 3 independent experiments performed in triplicates). Data are represented as means + S.E.M. *p* values were determined by ordinary one-way ANOVA. *p < 0.05, **p < 0.01, ***p < 0.0001

As *HMG20A* transcript levels were decreased in T2DM islets, we reasoned that lipids and/or glucose might alter its expression. Towards addressing this premise, we used our mouse model of 45% high fat diet (HFD)-induced obesity and pre-diabetes to assess the contribution of lipids. These mice exhibit a typical 20% increase in body weight, develop glucose intolerance with the concomitant increases in insulin and leptin levels and decreased adiponectin levels^25^. Although these mice developed insulin resistance, glycaemia is normal due to increased insulin levels (Fig. 2a, b). Under these conditions, HMG20A protein levels were not significantly changed in islets as compared to levels in chow-fed control mice (Fig. 2c). In contrast, short-term exposure of INS-1E cells to palmitate resulted in a transient reduction in HMG20A transcript levels (Fig. 2d).

**Fig. 3:**
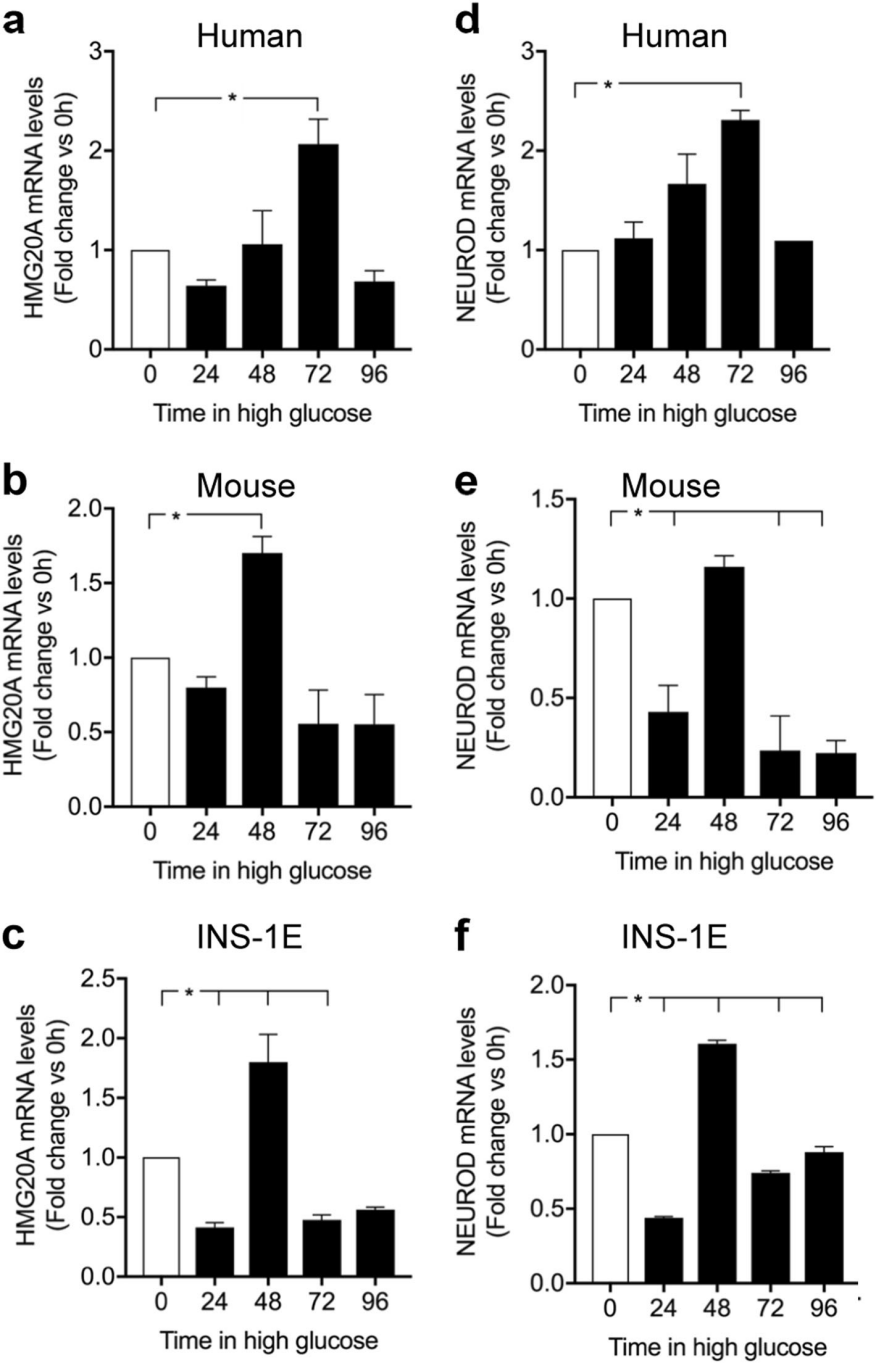
HMG20A is regulated by glucose. **a–c** *HMG20A* and **d–f** *NEUROD* mRNA levels were assessed by qPCR in human (*n* = 3 donors performed in triplicates) and mouse islets (three independent preparations of pooled islets from three different isolation) as well as INS-1E cells cultured in 24 mM glucose for 24, 48, 72, or 96 h (*n* = 3 independent experiments of all time points executed in triplicates). Data are represented as means + S.E.M. *p* values were determined by ordinary one-way ANOVA. *p < 0.05, compared to the control 0 time point

**Fig. 4:**
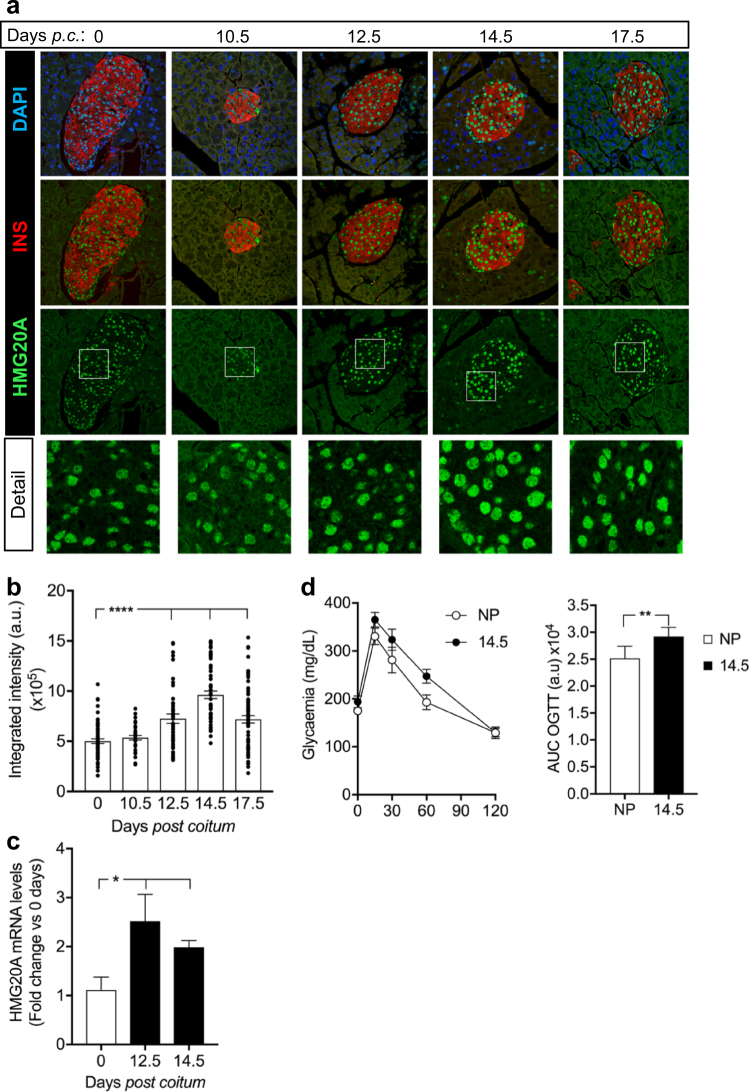
HMG20A is transiently increased in mouse islets during pregnancy. **a** Representative images of pancreatic sections from pregnant mice co-stained for HMG20A (green) and INSULIN (INS) (red). Nuclei were stained using DAPI (blue). Magnification 40×. White boxes define areas enlarged in panels below. p.c. post coitum. **b** Quantification of integrated fluorescence intensity for HMG20A in beta cells of islets from pregnant mice (*n* = 3 mice per time point with 35-71 islets counted per time point). Data are depicted as dot plots with means ± S.E.M. **c** HMG20A mRNA levels in islets from pregnant mice (*n* = 5 mice) at 12.5 and 14.5 days of pregnancy. **d** Oral glucose tolerance test (OGTT) and area under the curve (AUC) analysis of non-pregnant (NP) and pregnant (day 14.5 of pregnancy; D14.5) mice (NP, *n* = 8; D14.5, *n* = 6). Data are represented as means ± S.E.M. *p* values were determined by the unpaired two-tailed Student´s *t*-test (**d** for AUC and **c**) or ordinary one-way ANOVA with Dunnet’s multiple comparison test (**b**). *p < 0.05, **p < 0.01, ****p < 0.0001, compared to the control 0 time point

We next assessed the direct action of high glucose on *HMG20A* levels in isolated islets. In human islets, *HMG20A* expression levels were transiently increased by approximately twofold at 72 h returning to basal levels by 96 h (Fig. 3a). A similar transient induction pattern, albeit attaining maximum levels at 48 h, was also observed in mouse islets and in INS-1E beta cells (Fig. 3b,c). Interestingly, a significant decrease was detected INS-1E cells at 72 h of high glucose exposure (Fig. 3c). It is noteworthy that expression levels of *NEUROD*, a bona fide target of HMG20A, mirrored those of *HMG20A* in human islets as well as in INS-1E (Fig. 3d,f), following the same tendency in mouse islets (Fig. 3e). In order to satisfy metabolic demand by fetus and mother, glucose metabolism is altered during pregnancy inducing metabolic stress that, if ill adapted, will lead to gestational diabetes. We therefore pondered whether *Hmg20a* expression levels were modulated during pregnancy. Consistent with this premise, transcript and protein levels of the *Hmg20a* gene were increased reaching a peak of maximum signal at 12.5 and 14.5 days post coitum, respectively, and thereafter declining (Fig. 4a–c). Pregnant females displayed mild glucose intolerance at 14.5 correlating with the peak in HMG20A protein levels (Fig. 4d). Taken together, these data suggest that increased glucose levels both in vitro and in vivo stimulate HMG20A expression.

### The rs7119 SNP variant within the 3′ untranslated region (UTR) of human *HMG20A* reduces activity of a luciferase reporter gene

**Fig. 5:**
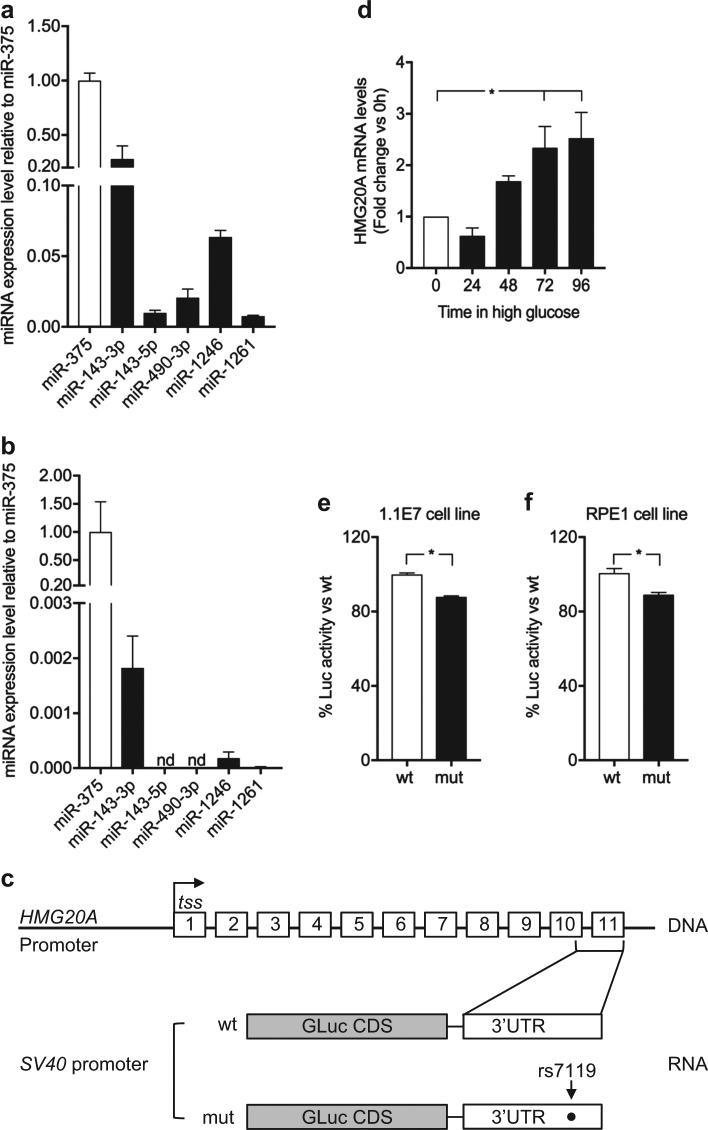
The rs7119 SNP within the *HMG20A* 3′ UTR decreases protein levels. Expression profile of putative miRNAs binding to the “mut” allele of the HMG20A transcript in **a** human 1.1E7 cells and **b** mouse islets. Data are presented respect to miR375 expression levels. **c** Schematic representation of the *HMG20A* gene depicting the promoter region (*HMG20A* promoter), transcription starting site (tss) and 11 exons. Gaussia luciferase (GLuc) reporter constructs harboring the 3′ UTR corresponding to either wild type (wt) or rs7119 mutant (mut) allele under *pSV40* promoter are shown below. **d** *HMG20A* mRNA levels assessed by qPCR in the human pancreatic beta cell line 1.1E7 cultured in 24 mM glucose for 24, 48, 72, or 96 h (*n* = 5 independent experiments performed in triplicates). **e** Human pancreatic beta 1.1E7 and **f** RPE1 cell lines were transfected with the reporter construct bearing either wt or rs7119 mut variant of the *HMG20*A 3′ UTR. Both the Gaussia Luciferase activity and SEAP activity were determined 24 h post transfection (*n* = 3 independent experiments). Data are represented as means + S.E.M. *p* values were determined by ordinary one-way ANOVA (**d**) or unpaired Student's *t*-test (**e**, **f**). *p < 0.05

Although GWAS have highlighted a panoply of SNPs correlating with T2DM, few have been assigned a functional consequence to the associated gene. Towards addressing this question, we assessed whether the human *HMG20A* SNPs associated to T2DM may impact HMG20A levels. Of particular interest is the SNP rs7119 located in the 3′ UTR of the *HMG20A* transcript that is encoded in part by exon 10 and the entirety of exon 11 (Fig. 5c). The SNP rs7119 causes a “C” (reference allele “wt”) to “T” (“mut” allele) substitution. In silico analysis of the *HMG20A* 3′UTR revealed the binding site of a single miRNA (miR-571) to the wt allele whereas the “mut” allele results in the ablation of this miRNA binding site (Table 1). Interestingly expression of miR-571 was not detected in mouse islets as compared to the highly expressed miR375 (data not shown). Notwithstanding, the “mut” allele generates binding sites for six alternative miRNAs (Table 1) of which four, miR-143 (3p and 5p), miR-490-3p, miR-1246, and miR-1261, were expressed in the human 1.1E7 pancreatic cell line (Fig. 5a). In contrast mouse islets only expressed miR-143 and very low levels of miR-1246 (Fig. 5b). The latter findings suggest that the “mut” variant may alter expression of the *HMG20A* transcript due to the action of those miRNAs. To assess this premise, we generated two Gaussia luciferase (Gluc) reporter constructs harboring either the “wt” or “mut” 3′ UTR of the human *HMG20A* transcript. Expression of these constructs was under the transcriptional regulation of the SV40 basal promoter (Fig. 5c). Transient transfections were then performed in the human pancreatic beta 1.1E7 and retinal pigmented epithelial RPE1 cell lines. The rational of using human cell lines was to maintain species-specific effects targeting the human 3′ UTR of the *HMG20A* transcript, which is distinct than that of the mouse *Hmg20a* transcript. We initially confirmed that glucose also stimulated *HMG20A* expression in 1.1E7 beta cells (Fig. 5d). The “mut” variant displayed significantly less Gluc activity as compared to the construct bearing the “wt” variant 48 h post transfection in either cell lines (Fig. 5e, f). These results suggest that the diabetes-linked SNP rs7119 alters expression of the human *HMG20A*.

**TABLE 1:** In silico identification of miRNAs binding sites within the rs7119 SNP located in the 3′UTR of HMG20A

“C” reference alelle	“T” risk allele
(wt)	(mut)
hsa-miR-571	hsa-miR-1261
	hsa-miR-1257
	hsa-miR-1246
	hsa-miR-586
	hsa-miR-490 (5p, 3p)
	hsa-miR-143 (5p, 3p)

### HMG20A regulates the expression of beta cell genes involved in metabolism-secretion coupling and beta cell maturity

**Fig. 6:**
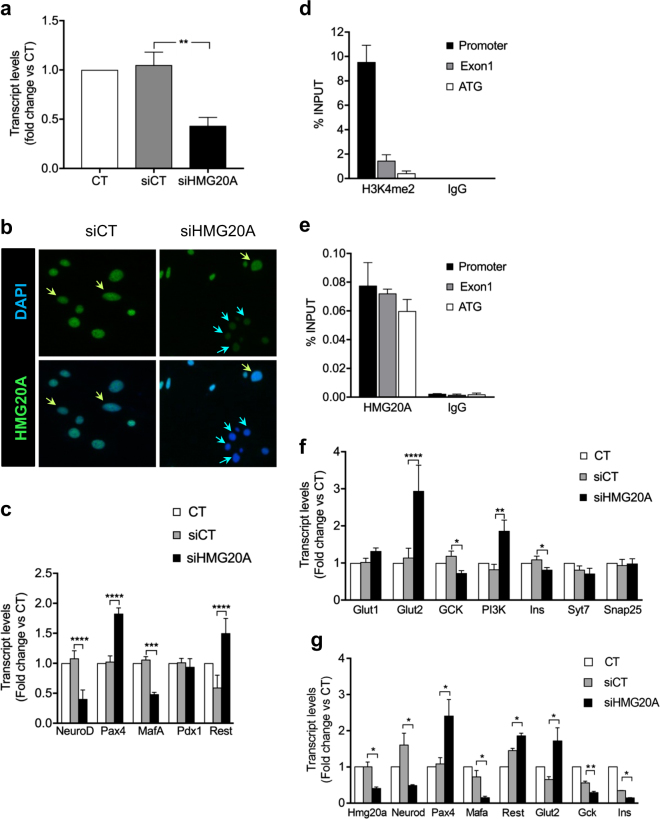
HMG20A regulates metabolism-secretion coupling genes in INS-1E cells. **a*** Hmg20a* was silenced by specific siRNA in INS-1E cells (*n* = 6 independent experiments, each performed in triplicates). **b** Representative images of INS-1E cells immuno-stained for HMG20A (green) and DAPI (blue) confirming decreased protein level after treatment with siHMG20A. Magnification 40×. **c** Beta cell development and maturity genes, as *Neurod*, *Pax4*, *Mafa, Pdx1*, and *Rest* transcripts levels after silencing of *Hmg20a* in INS-1E cells (*n* = 4 independent experiments performed in triplicates). **d** H3K4m2 and **e** HMG20A occupancy of promoter, exon 1 and ATG regions of the *Pax4* gene after chromatin immunoprecipitation using anti-H3K4m2 or anti-HMG20A antibodies, with IgG as a control for non-specific interactions (*n* = 4 independent experiments performed in triplicates). **f** Beta cell metabolism-secretion coupling genes transcripts levels after silencing of *Hmg20a* in INS-1E cells (*n* = 4 independent experiments, performed in triplicates). **g** Alteration in the expression of key genes was confirmed in INS-1E cells using a second siHMG20A (*n* = 4 independent experiments performed in triplicates). Data are represented as means + S.E.M. *p* values were determined by one-way ANOVA with Tukey’s multiple comparisons test (**a, c, f, g**). *p < 0.05, **p < 0.01, ***p < 0.001, ****p < 0.0001

To determine the specific contribution of HMG20A to islet beta cell function and the impact of its reduced levels in T2DM islets, we silenced *HMG20A* expression by RNA interference and then assessed transcript levels of key factors involved in beta cell identity and function. A 60% depletion of *Hmg20a* in INS-1E cells (Fig. 6a, b) resulted in a concomitant 60% inhibition of *NeuroD* transcript levels (Fig. 6c). *Mafa*, which is essential to maintain the beta cell phenotype, was decreased by 50% whereas *Pdx1* was unaltered. Interestingly, *Pax4* as well as *Rest* were increased by 1.8- and 1.5-fold, respectively, subsequent to *Hmg20a* silencing (Fig. 6c). We previously demonstrated that chronic overexpression of *Pax4* leads to loss of beta cell identity characterized by decreased *Mafa* and *Insulin* expression^23^. We thus, pondered whether HMG20A could directly regulate *Pax4* thereby maintaining its expression within a range that would not interfere with beta cell function. To validate this premise, chromatin immunoprecipitation (ChIP) experiments were performed using the islet specific *Pax4* gene promoter^24^. As expected H3K4me2, a marker for active promoters and enhancers specifically occupied this region (Fig. 6d)^26^. More importantly, HMG20A also occupied the *Pax4* gene promoter region as well as exon 1 while control anti-IgG exhibited no binding (Fig. 6e). Since NEUROD and MAFA are involved in metabolism-secretion coupling in beta cell, genes implicated in this process were assessed after *Hmg20a* silencing. *Glucokinase* (*Gck*) and *Insulin* transcript levels were significantly repressed by 30% (Fig. 6f). In contrast, *Pi3k* and *Glut2* levels were increased 1.8 and 2.6-fold, respectively (Fig. 6f). Transcript levels of *Syt7* and *Snap25*, two factors involved in insulin granule membrane docking and fusion were not altered in *Hmg20a*-repressed INS-1E cells (Fig. 6f). The modulation of key HMG20A target genes was validated using a second independent siHMG20A (Fig. 6g). Taken together our results indicate that HMG20A coordinates expression of genes that will establish the mature beta cell phenotype.

### Glucose-stimulated insulin secretion is impaired by *Hmg20a* depletion

**Fig. 7:**
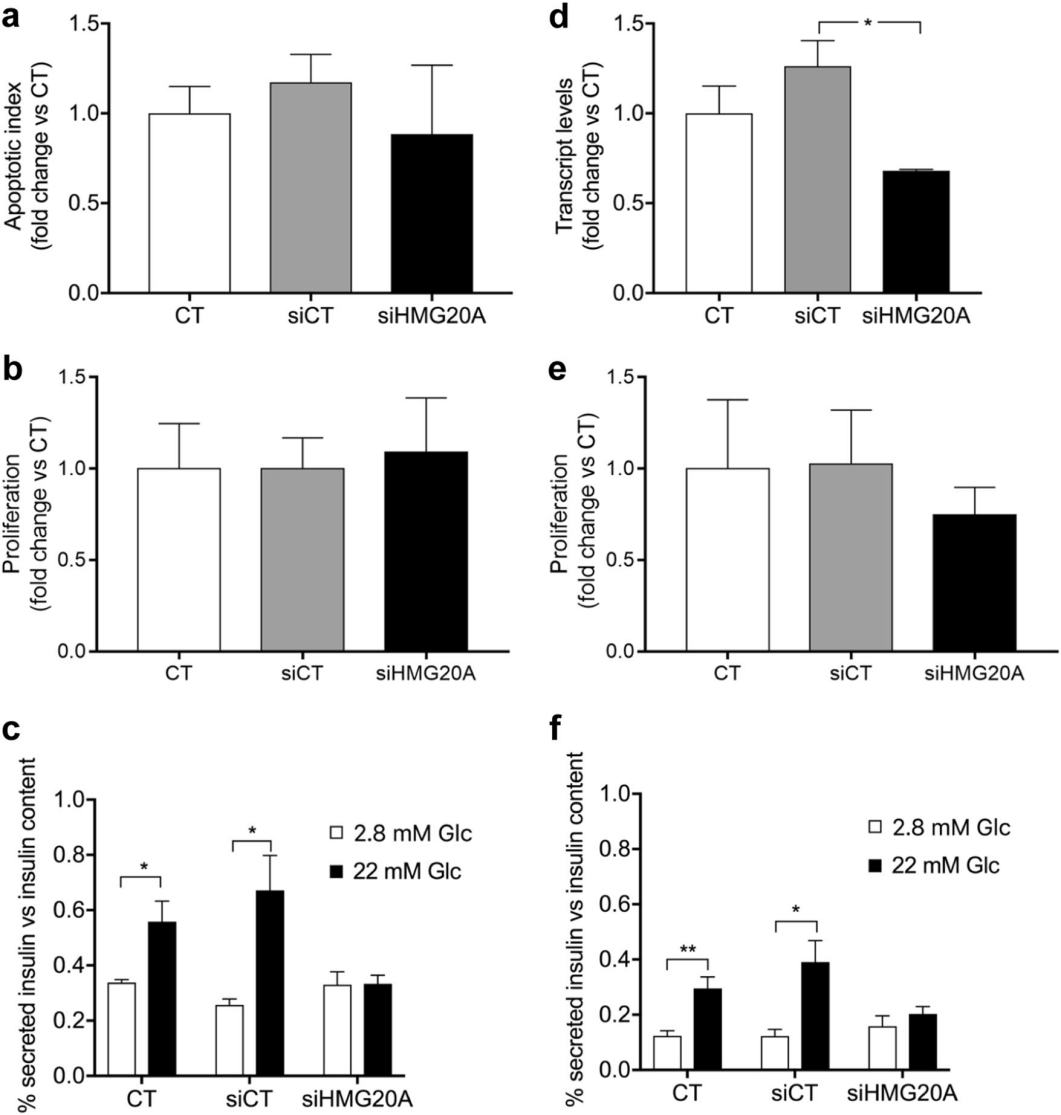
*Hmg20a* silencing impairs insulin secretion in INS-E1 cells as well as in mouse islets without affecting cell death or proliferation. **a** Cell death (*n* = 3), **b** proliferation (*n* = 3), and **c** glucose-induced insulin secretion (GSIS, *n* = 4) were assessed subsequent to siRNA-mediated HMG20A depletion in INS-1E cells. **d** Effect of siRNA-mediated HMG20A depletion on **e** cell proliferation (*n* = 6 islet preparations) and **f** GSIS in mouse islets (*n* = 6 islet preparations). Data are represented as mean + S.E.M. *p* values were determined by unpaired two-tailed Student's *t*-test. *p < 0.05, **p < 0.01

We next assess the cellular and functional consequences of *Hmg20a* depletion in beta cells. Neither cell death nor proliferation was altered by HMG20A depletion in INS-1E cells (Fig. 7a, b). In contrast, glucose-stimulated insulin secretion (GSIS) was blunted in these cells (Fig. 7c). Similarly, subsequent to a 40% depletion of *Hmg20a* in mouse islets (Fig. 7d), cell proliferation was unaltered while GSIS was drastically decreased as compared to control islets (Fig. 7e, f). Taken together, *Hmg20a* expression is essential for insulin secretion.

## Discussion

The overall aim of the current study was to assign a functional role of the putative diabetes-linked *HMG20A* gene to islet physiology, and determine the pathophysiological consequence of the rs7119 SNP associated with this disease. We find that: (1) *HMG20A* is expressed in pancreatic islet beta cells, (2) HMG20A levels are decreased in T2DM islets, (3) high glucose stimulates HMG20A expression, (4) the T2DM-associated SNP within the 3′ UTR of the HMG20A transcript decreases luciferase activity, (5) HMG20A regulates key genes in beta cell function and maturity, and (6) HMG20A depletion impairs GSIS. Therefore, we provide first evidence that *HMG20A* is a key regulator of mature beta cell function.

Our results indicate that variations in glucose levels are a main rheostat of islet HMG20A expression, which in turn appears to modulate islet adaptive measures by increasing insulin biosynthesis. The latter premise is supported by our findings that *NEUROD* expression levels mirrored those of *HMG20A*, being stimulated by high glucose and inhibited under siRNA-mediated *Hmg20a* downregulation. Mutations in the *NEUROD* gene predispose individuals to develop maturity onset diabetes of the young (MODY6)^17^ while adult mouse islets lacking *Neurod* respond poorly to glucose^27^. We also found reduced expression levels of *Mafa* as a consequence of *Hmg20a* depletion. This transcription factor along with NEUROD activates expression of the *Gck* and *Insulin* gene^27–29^, consistent with reduced levels of both transcripts in Hmg20A-silenced cells. Intriguingly, *Glut2* was increased following *Hmg20a* repression perhaps as a compensatory mechanism to increase cellular glucose influx due to decreased GCK-mediated phosphorylation and glycolysis^30^. Thus, the coordinated regulation of *NeuroD* and *Mafa* by HMG20A may be a key molecular hub essential for beta cell function and adaptation to physiological stress such as during pregnancy in which glucose metabolism is altered. Interestingly, although HMG20A was repressed by palmitate *in vitro*, expression levels were not significantly altered in islets of a mouse model of high fat diet-induced obesity and pre-diabetes. Thus, it is tempting to speculate that *in vivo*, glucose and lipids may have opposite effect on HMG20A expression nullifying any significant increased in expression, which may in long-term hinder islet adaptation, precipitating beta cell dysfunction and T2DM. The latter hypothesis may rationalize the observed association of HMG20A risk variants with T2DM in obese cases^8^.

Our study also identifies the *Pax4* gene as a direct target repressed by HMG20A. Although the *Pax4* gene promoter was shown to interact with REST^15^, the absence of this “disallowed” gene in beta cells suggest that HMG20A acts independent of the LSD1–coREST complex that requires REST to be recruited onto DNA^14^. Rather, our results are in agreement with recent findings that HMG20A in cohort with LSD1 is sufficient to repress *Snail1* and induce TGF beta-triggered EMT, demonstrating a mechanism independent of REST as well as the capacity of HMG20A for binding directly to DNA^18^. Notwithstanding*, Hmg20a* silencing also evoked REST re-expression indicating that in cohort with PAX4, these two factors may promote de-differentiation of beta cells conveying increased survival under unfavorable physiological conditions such as chronic hyperglycemia.

To date, regulation of the endogenous *Pax4* gene in mature islets conveying protection and adaptation in response to physiological or pathophysiological conditions has remained obscure. Our data suggest that HMG20A may be an important epigenetic regulator of *Pax4* gene expression that will dictate the faith of beta cells under stress conditions such as in T2DM. In accord with this premise, we previously demonstrated that PAX4 expression was increased in islets derived from T2DM patients^31^. We now demonstrate that HMG20A levels are repressed in T2DM islets. These combined human data are in line with increased expression of *Pax4* subsequent to *Hmg20a* silencing in either islets or INS-1E cells without altering apoptosis and proliferation. Accumulating evidence suggest that the main characteristic event in T2DM is not massive beta cell death but rather beta cell de-differentiation^32^. We previously demonstrated that chronic expression of *Pax4* is implicated in this process through downregulation of *Mafa* and *Insulin* expression^33^. Our current data substantiate these findings as we detected a decrease in *Mafa* expression after *Hmg20*a silencing. Thus, the role of HMG20A may be to supress *Pax4* transcription thereby permitting expression of *Mafa* as well as *Insulin*. Such cross talk between the two factors is highlighted also during pregnancy. Indeed, we previously found a peak of *Pax4* expression and beta-cell proliferation at 10.5 days and 12.5 days of pregnancy respectively^24^. Herein, we now find that HMG20A expression in islets is increased at 14.5 days of pregnancy, just after the peak of proliferation and correlating with decreased *Pax4* and increased *Mafa* expression levels. These findings are consistent with the premise that beta cells after proliferation have to re-differentiate to reach their metabolic mature phenotype and adequately secrete insulin, a process requiring MAFA^34^. A similar regulatory cross talk between HMG20A and PAX4 may also be operative in early stages of hyperglycemia, which is characterized by active beta cell adaptation. Nonetheless, long-term exposure may favor de-differentiation and survival (without altering proliferation) to the detriment of function, as we observed *in vitro* in which *HMG20A* expression was transiently increased but then inhibited by glucose. Although further studies will be needed to specify the mechanism by which HMG20A regulates those islet-enriched genes, our work provides the basis for the involvement of a non-mutually exclusive cross regulation of HGM20A and PAX4 in islet/beta cell physiology.

A significant finding of our study is the potential decreased in HMG20A levels associated with the diabetes-linked rs7119 SNP within the 3′ UTR of the transcript. This SNP opens the putative binding site of several new miRNAs as compared to the normal wild type allele. Although a previous study has linked T2DM-associated genes to islet-expressed miRNA^35^, our study, to the best of our knowledge, provides first evidence that a diabetes-linked SNP alters the putative binding site for miRNAs resulting in the deregulate repression of the target transcript. Further indication for a dysfunctional role of the rs7119 variant in aberrant HMG20A repression was highlighted by the expression of SNP-associated miRNAs, the most abundant being miR-143 and 1246, in human 1.1E7 cells and mouse islets. In contrast, miR-571 associated with the wild type allele miRNA binding site was not express suggesting the potential absence of post-transcriptional regulation conveyed by the normal allele. Although scarce details are available on the role of miR-143 and 1246 in islet function, miR-143 was shown to be among the ten most abundant miRNAs expressed in human islets beta cells whereas miR-1246 was predominantly expressed in other islet cell types^35^. Elsewhere, miR-143 expression was found to be essential for human pre-adipocyte differentiation partly through repression of its target gene ERK5 involved in cell growth and proliferation^36^. Although it remains to be validated, the potential serendipitous binding of miR-143 to the HMG20A rs7119 gene variant may induce aversely de-differentiation through activation of *Pax4* and *Rest*.

The overall data presented in this work clearly describes a functional link between HMG20A and islet physiology. HMG20A regulates metabolism-secretion coupling genes in beta cells and could be also involved in beta cell plasticity through PAX4 and REST, regulating phenotypic changes needed for beta cells in order to respond to different physiological situations as pregnancy and obesity. Failure on those HMG20A-mediated processes could lead to the development and establishment of T2DM. As consequence, our study opens a venue to consider targeting of HMG20A expression and/or regulation as potential therapies for T2DM.

## Materials and methods

### Animals

The experimental mouse procedures were approved by the Institutional Animal Care Committee of the Andalusian Center of Molecular Biology and Regenerative Medicine (CABIMER) and by the ethic committee of the University of Malaga, Biomedical Research Institute of Málaga (IBIMA) and performed according to the Spanish law on animal use RD 53/2013. Animal studies were performed in compliance with the ARRIVE guidelines^37^. Mice were housed in ventilated plastic cages and maintained on a 12-h light–dark cycle with ad libitum access to pelleted chow and water. C57BL/6J of both genders were used to obtain pancreatic islets and other tissues (liver, adipose tissue, skeletal muscle, and brain) and females for pregnancy studies. For diet-induced obesity studies, 8-weeks-old male C57BL/6J mice (Janvier Labs, Saint-Berthevin Cedex, France) were housed in individual cages under a 12 h light/dark cycle (8:00 pm light off) in a room with controlled temperature (21 ± 2 °C) and humidity (50 ± 10%). For induction of obesity, groups of ten mice were fed a high fat diet (D12451 Research Diets Inc., New Brunswick, NJ, USA), containing 45% of Kcal from saturated fat, for 15 weeks. In parallel, groups of ten age-matched mice were fed a control diet containing 10% of Kcal from fat (D12450 Research Diets Inc.). Circulating glucose levels were measured from tail vein blood samples using an Optium Xceed glucometer (Abbott Scientifica SA, Barcelona, Spain). Insulin tolerance tests (ITT) and glucose tolerance test (OGTT) were performed as previously described^38^. At 15 weeks, mice were sacrificed by cervical dislocation and pancreas extracted for histological processing.

### Pancreatic islet and cell line cultures

Mouse pancreatic islets were isolated from 2 to 4-months-old C57BL/6J mice by intraductal collagenase as previously described^24,39^ . After isolation, mouse islets were cultured in 5.5 mM glucose/RPMI 1640 medium (Life Technologies, Madrid, Spain) supplemented with 10 % Fetal bovine serum (FBS, Sigma-Aldrich, Madrid, Spain), 100 U/ml penicillin, 100 μg/ml streptomycin (Sigma-Aldrich), 2 mM l-glutamine (Life Technologies), 1 mM sodium pyruvate (Sigma-Aldrich), 50 μM β-mercaptoethanol (Life Technologies), and 10 mM HEPES (Life Technologies) prior to different experimental treatments. Islets from non-diabetic or T2DM organ donors were obtained in Pisa or purchased from Tebu-Bio (Barcelona, Spain). After reception, human islets were cultured in CMRL-1066 (Gibco) medium containing 5.5 mM glucose and supplemented with 10% FBS, 100 U/ml penicillin, 100 μg/ml streptomycin, 2 mM l-glutamine, and 100 μg/ml gentamycin. To assess glucose influence in HMG20A expression, both human and mice islets were cultured in normal (5.5 mM) or high (24.4 mM) glucose concentrations for 24, 48, 72, and 96 h, and lately harvested for RNA extraction.

The rat insulinoma INS-1E beta cell line (kindly provided by Dr. P Maechler, Geneva, CH) was cultured between passages 50–90 and maintained in 11.1 mM glucose/RPMI 1640 medium (Life Technologies) supplemented with 10% FBS, 100 U/ml penicillin, 100 μg/ml streptomycin, 2 mM l-glutamine, 1 mM sodium pyruvate, 50 μM β-mercaptoethanol, and 10 mM HEPES. Following overnight glucose deprivation (3 mM), cells were cultured in 11.1 mM or 24.4 mM glucose RPMI 1640 medium for 24, 48, 72, and 96 h. Alternatively INS-1E cells were also exposed to 0.5 mM palmitate for up to 96 h. Cells were harvested at each time point for RNA extraction.

The human retinal pigmented epithelial RPE1 (ATCC, CRL-4000) and the islet beta 1.1E7 cell lines (The European Collection of Authenticated Cell Cultures) were cultured in F-12 Ham and RPMI media, respectively, prior to luciferase reporter assays. The 1.1E7 cell line was also cultured in the presence of either 11.1 or 24.4 mM glucose for up to 96 h. RNA was extracted at 24, 48, 72, and 96 h post treatment.

### RNA interference

Mouse pancreatic islets and INS-1E cells were cultured as previously described^40^ and transfected with either 50 μmol of two independent *HMG20A* small interfering (si)RNAs (5′-AGGCAAAUCUCAUAGGCAA-3′ and 5′-UCACAAGGAUGUUGGGCAA-3′) or scramble siRNA (Sigma-Aldrich) using Oligofectamine (Life Technologies). Samples were processed for RNA, immunofluorescence, GSIS, cell proliferation and death assessment 72 h after transfection.

### RNA extraction and quantitative-PCR expression analysis

Qiagen RNeasy Micro and Mini kits (Qiagen, Madrid, Spain) were used for the extraction of total RNA from the different tissues, islets and cell line samples. After quantification of the RNA concentration using a NanoDrop 1000 Spectrophotometer (Wilmington, DE, USA), single-stranded cDNA was synthesized with the Superscript II First-Strand cDNA synthesis kit (Invitrogen, Carlsbad, CA, USA) and Anchored OligodT as primers (Sigma-Aldrich). Quantitative PCR was performed on a 7500 Real-Time PCR System (Applied Biosystems). Gene-specific primers (*HMG20A, NEUROD1, PDX1, PAX4, REST, GLUKOKINASE, INSULIN, PI3K, MAFA, GLUT1, GLUT2, SNAP25*, and *SYT7*) were designed using Power Primer3 (Supplementary Table 1, 2 and 3). Expression levels of the housekeeping genes *β-ACTIN* or *CYCLOPHILIN* were used for normalization. The relative gene expression was calculated using the 2^−ΔΔCt^ method^24^. For miRNA expression profiling, microRNAs were extracted from the 1.1E7 beta cell line and mouse pancreatic islets using the miRNAeasy kit (Qiagen) and cDNA synthesis was conducted using the Universal cDNA Synthesis Kit (Exiqon) as previously described^41^. ExiLENT SYBR Green Master Mix Kit and primers for each assay to perform quantitative-PCR (qPCR) were obtained from Exiqon.

### Immunofluorescence and immunohistochemistry studies

Dissected pancreases were fixed overnight in 4% paraformaldehyde at 4 °C and processed for embedding in paraffin at the Histology Core Facility, CABIMER. Pancreatic sections from non-pregnant/pregnant mice sacrificed at 10.5, 12.5, 14.5, and 17.5 days post coitum were analyzed by immunohistochemistry using specific antibodies against HMG20A, insulin, glucagon, and somatostatin (Supplemental Table 4). Counterstaining was performed with 5 μg/ml DAPI (Life Technologies) to stain the nuclei and slides were mounted using fluorescent mounting medium (DAKO). Images were acquired using either the epifluorescence (Leica AF6000, Leica, England) or confocal (Leica TCS SP5) microscopes. Expression of HMG20A in beta cells was quantified using Metamorph Analysis Software (Molecular Devices). Single frames were acquired using a confocal TCS SP5 microscope (Leica). Same illumination settings were applied for all the samples (image parameters including pinhole size, photomultiplier offset and gain, and laser intensity were first set for non-pregnant control samples, and then, the same settings were used for all conditions). For Image analysis/quantification, the integrated intensity values (∑ pixel intensity at region of interest) were measured as previously described^42–44^. Integrated intensity of nuclear HMG20A in islet cells co-expressing insulin were measured, and the average of intensity per islet was calculated. Three independent animals and an average of 50 islets were used for each time point. Pancreatic sections from control and HFD mice were performed and analyzed as previously described^25^.

### Luciferase 3′ UTR reporter assays

The effect of the 3′ UTR SNP rs7119, previously linked to T2DM was evaluated by luciferase reporter assays. 3′ UTR constructs containing the wt allele (reference allele) “C” or the DMT2-linked allele (“mut” allele) “T” cloned downstream of the luciferase gene were obtained from GeneCopoeia. Luciferase contained in these constructs is a secreted one; therefore luciferase activity can be measured in the medium. These constructs also have a secreted embryonic alkaline phosphatase (SEAP) gene driven by a constitutive promoter, used as internal control. The Secrete-Pair TM Dual Luminescence Assay Kit (GeneCopoeia) was used as luciferase reporter system according to manufacturer’s instructions. RPE1 and 1.1E7 cell lines were chosen for transfection studies using those constructs. Both are cell lines of human origin and from different tissues. RPE1 are cells from retina pigmentosum ephitelia^45^ meanwhile 1.1E7^46^ is a human beta cell line. The RPE1 and 1.1E7 cell lines were transfected and luciferase activity was measured 48 h after in the collected culture media.

### Glucose-stimulated insulin secretion assays

Insulin secretion in response to 2.8 mM or 22 mM glucose was measured in static incubations as previously described^47,48^ in both mouse pancreatic islets and INS-1E cells. Following the incubations, supernatants were collected and islets and cells were lysated with HCl-ethanol to obtain the insulin content. Insulin from supernatants and cell contents was measured by ELISA (Mercodia, Uppsala, Sweden). All experiments were run in triplicate.

### Cell death and proliferation

Cell death (apoptosis) and proliferation was measured using either the Cell Death Detection ELISA kit or the 5-Bromo-2′-deoxy-uridine labeling and detection kit (Roche Diagnostics, Madrid, Spain) as described by the manufacturer.

### ChIP assays

ChIP assays were performed as previously described^12,18^. INS-1E cells were treated with 1% formaldehyde for 15 min at 37 °C for crosslinking. Chromatin was sonicated to an average fragment size of 400 to 500 bp using the Diagenode Bioruptor. Immunoprecipitations were performed using the following reagents: Dynabeads Protein A (Invitrogen), rabbit polyclonal anti-HMG20A (Sigma-Aldrich) and rabbit monoclonal to Histone H3 (di methyl K4) (Abcam). Rabbit IgG (Sigma) was used as a control for non-specific interactions. Input was prepared with 10% of the chromatin used for immunoprecipitation. Quantification of immunoprecipitated DNA was performed by real-time PCR with the Applied Biosystems 7500 FAST real-time PCR system, using Applied Biosystems Power SYBR green master mix. Each sample was quantified in triplicate. Provided data are the average of at least three independent experiments.

### Statistical analysis

The Ruth Lenth's power of analysis was applied to the different animal models to ensure that adequate numbers of animals had been studied to detect significant changes. Results are expressed as mean ± SEM (line plots as a function of time) or as mean + SEM (bar graphs). Statistical analyses were performed using the GraphPad Prism software version 7 (GraphPad Software, La Jolla, USA).

## Electronic supplementary material

Supplemental material
