# Supplementary material for: The type 2 diabetes-associated HMG20A gene is mandatory for islet beta cell functional maturity
Source: Cell Death Dis. 2018 Feb 15;9(3):279. doi: 10.1038/s41419-018-0272-z (PMC5833347; doi:10.1038/s41419-018-0272-z)
Supplement: Supplementary file 1 — Supplemental material [file 41419_2018_272_MOESM1_ESM.docx]

**SUPPLEMENTARY MATERIALS**

**SUPPLEMENTARY TABLE 1**: List of rat primers used in this study.

| **Gene name** | **ID** | **Forward primer** | **Reverse primer** |
| --- | --- | --- | --- |
| High mobility group 20A | Hmg20a | AACTGAGACAGGGCAGAGGA | ACTTCAGCAGAGGGGCTGTA |
| Neuronal differentiation 1 | Neurod | GAACCATCCACCGAGTTTGAA | AAGATTGATCCGTGGCTTTGG |
| Pancreatic and duodenal homeobox 1 | Pdx1 | CCGCGTTCATCTCCCTTTC | CTCCTGCCCACTGGGTTTT |
| Paired box 4 | Pax4 | TGGACACCCGACAGCAGAT | CTTAAGGCTCCGTGAGATGTCA |
| Glucokinase | Gck | ACCTGAGTGTTGGAGATGATTC | CCGAGTGGCTTACAGTTCTG |
| Insulin | Ins | CCCACACACCAGGTACAGAGC | CAGCACCTTTGTGGTCCTCA |
| Phosphotidylinositol 3 | Pi3k | TGGGACCTTTTTGGTACGAGA | AGCTAAAGACTCATTCCGGTAGT |
| MAF bZIP transcription factor A | Mafa | CAGCAGCGGCACATTCTG | CCGCCAACTTCTCGTATTTCTC |
| Glucose transporter 1 | Glut1 | TGTGCTCATGACCATCGC | AAGGCCACAAAGCCAAAGAT |
| Glucose transporter 2 | Glut2 | CATTGCTGGAAGAAGCGTATCAG | GAGACCTTCTGCTCAGTCGACG |
| Synaptosomal-associated protein 25 | Snap25 | TCCCTGGAAAGCACCCGTCG | CCGTTCATCCACCACACGGGC |
| Synaptotagmin VII | Syt7 | GTAGCGTTTGCCCAGTTTGC | CCTTCAGCCTTAGCGTCACT |
| RE-1 silencing trancription factor | Rest | ACACAGGAGAACGCCCTTAT | GAGGCCACATAATTGCACTG |
| Beta Actin | β-Actin | CCAGTGGTACGACCAGAGGC | CGTAGCCATCCAGGCTGTGT |

**SUPPLEMENTARY TABLE 2**: List of mouse primers used in this study.

| **Gene name** | **ID** | **Forward primer** | **Reverse primer** |
| --- | --- | --- | --- |
| High mobility group 20A | Hmg20a | AACCAACCCAGAGTTTGTGG | TTGCTCATCTTCAGGCCTTT |
| Neuronal differentiation 1 | Neurod | CTCGGACTTTCTTGCCTGAG | TTTCAAAGAAGGGCTCCAGA |
| Beta Actin | β-Actin | TCCTGTGGCATCCACGAAACTACA | ACCAGACAGCACTGTGTTGGCATA |

**SUPPLEMENTARY TABLE 3**: List of human primers used in this study.

| **Gene name** | **ID** | **Forward primer** | **Reverse primer** |
| --- | --- | --- | --- |
| High mobility group 20A | HMG20A | GCATGAAGATGAGCAACGAA | GCTCATTCATGAACCGAACA |
| Neuronal differentiation 1 | NEUROD | GTTCTCAGGACGAGGAGCAC | CTTGGGCTTTTGATCGTCAT |
| Cyclophilin | CYCLO | CCATTTGTGTTGGGTCCAGC | TACGGGTCCTGGCATCTTGT |

**SUPPLEMENTARY TABLE 4**: List of antibodies used in this study.

| **Primary antibodies** | **Host** | **Dilution** | **Supplier** | **Catalog number** |
| --- | --- | --- | --- | --- |
| HMG20A | Rabbit | 1:100 | Sigma-Aldrich | HPA008126 |
| Insulin | Mouse | 1:500 | Sigma-Aldrich | I2018 |
| Glucagon | Mouse | 1:200 | Sigma-Aldrich | A944 |
| Somatostatin | Goat | 1:100 | Santa Cruz Biotechnology | SC-7819 |
| **Secondary antibodies** | **Host** | **Dilution** | **Supplier** | **Catalog number** |
| Alexa fluor 568 goat anti-mouse | Goat | 1:800 | Thermo Fisher Scientific | A11004 |
| Alexa fluor 488 goat anti-rabbit | Goat | 1:800 | Thermo Fisher Scientific | A11008 |

**SUPPLEMENTARY TABLE 5**: Characteristics of non-diabetic (ND) and type 2 diabetic (T2DM) donors. BMI, body mass index; CVD, cardio-vascular disease.

| ND | Age (yrs) | Gender | BMI (Kg/m^2^) | Cause of death |
| --- | --- | --- | --- | --- |
| Donor #1 | 79 | F | 27.5 | CVD |
| Donor #2 | 62 | M | 34.8 | CVD |
| Donor #3 | 39 | M | 32.7 | CVD |
| Donor #4 | 77 | F | 35.4 | CVD |
| Donor #5 | 47 | M | 23.5 | Trauma |
| Donor #6 | 48 | F | 22.5 | CVD |
| Donor #7 | 47 | M | 37.8 | CVD |
| T2DM | Age (yrs) | Gender | BMI (Kg/m^2^) | Cause of death |
| Donor #8 | 79 | F | 29.3 | Trauma |
| Donor #9 | 72 | M | 32.4 | CVD |
| Donor #10 | 78 | M | 26 | Trauma |
| Donor #11 | 76 | M | 24.5 | CVD |
| Donor #12 | 53 | F | 29.4 | CVD |
| Donor #13 | 66 | M | 23.1 | CVD |
| Donor #14 | 52 | M | 34.9 | CVD |
